# Bilingualism affects audiovisual phoneme identification

**DOI:** 10.3389/fpsyg.2014.01179

**Published:** 2014-10-21

**Authors:** Sabine Burfin, Olivier Pascalis, Elisa Ruiz Tada, Albert Costa, Christophe Savariaux, Sonia Kandel

**Affiliations:** ^1^LPNC (CNRS UMR 5105) – Université Grenoble AlpesGrenoble, France; ^2^Universitat Pompeu FabraBarcelona, Spain; ^3^Institució Catalana de Recerca i Estudis AvançatsBarcelona, Spain; ^4^GIPSA-lab (CNRS UMR 5216) – Université Grenoble AlpesGrenoble, France; ^5^Institut Universitaire de France

**Keywords:** phonological deafness, bilinguals, monolinguals, audiovisual speech

## Abstract

We all go through a process of perceptual narrowing for phoneme identification. As we become experts in the languages we hear in our environment we lose the ability to identify phonemes that do not exist in our native phonological inventory. This research examined how linguistic experience—i.e., the exposure to a double phonological code during childhood—affects the visual processes involved in non-native phoneme identification in audiovisual speech perception. We conducted a phoneme identification experiment with bilingual and monolingual adult participants. It was an ABX task involving a Bengali dental-retroflex contrast that does not exist in any of the participants' languages. The phonemes were presented in audiovisual (AV) and audio-only (A) conditions. The results revealed that in the audio-only condition monolinguals and bilinguals had difficulties in discriminating the retroflex non-native phoneme. They were phonologically “deaf” and assimilated it to the dental phoneme that exists in their native languages. In the audiovisual presentation instead, both groups could overcome the phonological deafness for the retroflex non-native phoneme and identify both Bengali phonemes. However, monolinguals were more accurate and responded quicker than bilinguals. This suggests that bilinguals do not use the same processes as monolinguals to decode visual speech.

## Introduction

Having a conversation in a non-native language is a difficult task when our proficiency with that language is limited. To have a fluent conversation, we have to learn new vocabulary, syntax rules, and deal with new speech sounds. The present study examined whether the visual information provided by the articulatory gestures of the speaker enhances the identification of phonemes of other languages, especially those that do not exist in our phonological inventory. For example, the English phoneme /θ / does not exist in French. Native French speakers have difficulties identifying it and often confuse the /θ /-/f/ or /θ /-/s/ contrasts like in the words /θ In/ (*thin*), /fIn/ (*fin*), and /sIn/ (*sin*). So when they hear *thin*, they assimilate /θ / to the closest phoneme they know, in this case to /f/ or /s/ (Best, 1995). This example illustrates the phenomenon of “phonological deafness” (Polivanov, 1931). It refers to the difficulty or inability to identify phonemes that do not exist in the native phoneme inventory. When the non-native phoneme shares phonetic features with phonemes that exist in our phonological repertoire, we tend to confuse the non-native phoneme with the native one. This research examined how linguistic experience—i.e., the exposure to a double phonological code during childhood—affects the visual processes involved in non-native phoneme identification.

Phonological deafness results from perceptual narrowing processes. At birth, infants are capable of discriminating all phonological contrasts (Werker and Tees, 1984; Kuhl et al., 2006). This ability decreases progressively during the first year of life. As we become experts in the languages we hear in our environment we lose the ability to identify phonemes that do not exist in our native phonological inventory. Werker and Tees (1984) showed that 6–10 months old English-speaking babies could discriminate Salish and Hindi consonant phonemes that do not exist in English. At 10 months, their ability to discriminate these phonemes decreased significantly and almost disappeared at 12 months. The 12-months old children were phonologically deaf to these phonemes whereas Salish and Hindi-speaking infants of the same age could distinguish the phonemes perfectly well. These experiments tested the infants on auditory perception. A more recent study provided evidence for perceptual narrowing also in audiovisual speech. In Spanish, the English contrast /b/-/v/ (like in the words *ban* and *van*) does not exist. Phoneme /v/ does not exist in Spanish and is often assimilated to /b/ that does exist. Pons et al. (2009) presented this contrast audiovisually to English and Spanish-speaking 6 and 11 months infants. The results showed that both groups were audiovisually sensitive to the /b/-/v/ contrast at 6 months. At 11 months however, the Spanish-speaking babies lost this sensitivity but not the English-speaking ones. Although this decrease in phoneme discrimination abilities is a well-known phenomenon (see Best, 1995 for a review) and constitutes a real difficulty for second language learners, it is clear that we are all able to learn a non-native language after 12 months. A great majority of the world's population can communicate in several languages without having grown up in a multi-lingual environment (Altarriba and Heredia, 2008).

Most of us have experienced that to have a conversation in a non-native language is very difficult when we are not proficient with it. It becomes even more difficult if we cannot see the speaker's face, like when we are on the phone. This is likely due to the fact that on the phone we cannot see the articulatory movements of the speaker, which may provide visual cues on phoneme identity. A few studies presented data indicating that when having to deal with a non-native language, these visual cues may enhance performance (Davis and Kim, 2001; Hazan et al., 2006). Burfin et al. (2011) conducted an experiment that directly concerns phonological deafness. French native speakers had to identify the Spanish inter-dental fricative phoneme /θ /; that does not exist in French. The participants systematically identified /θ / as /f/ when the phonemes were presented auditorily. In other words, they were phonologically deaf to the /θ /-/f/ contrast. In an audiovisual presentation, where the participants could see the speaker producing the phonemes, /θ / was no longer confused with /f/. It was identified correctly up to 80–90%. This suggests that the participants used the visual cues provided by the speaker to overcome phonological deafness. This is in line with previous research presented by Navarra and Soto-Faraco (2007). They showed that Spanish-Catalan bilinguals who were Spanish dominant failed to distinguish the Catalan /e/-/ε / contrast (that does not exist in Spanish) in an audio-only presentation. In contrast, they could discriminate the phonemes in an audiovisual presentation. Taken together, these studies, carried out in several languages, reveal that the visual information on the speaker's articulatory movements can be very useful to overcome—at least partially—phonological deafness.

The visual information on the speaker's speech movements also seems to play a key role to discriminate languages, but the visual sensitivity depends on early linguistic exposure. Weikum et al. (2007) conducted an experiment in which 6 and 8-months old infants viewed silent videos of a bilingual French-English speaker telling a story either in French or English. One group of infants lived in an English monolingual environment whereas the other grew up in a French-English bilingual environment. The results indicated that all the 6 month-old infants could distinguish the French and English stories. At 8 months-old the English monolingual group could not distinguish the English and French stories. The 8 months-old bilinguals instead could distinguish them. Sebastian-Gallés et al. (2012) provided further data indicating that the infants' linguistic experience at birth is determinant for developing the visual sensitivity to language discrimination, irrespective of the languages they are exposed to. The authors presented the same French and English silent video stories to 8 months monolingual Spanish or Catalan and bilingual Spanish-Catalan infants that had never heard English or French before. The results revealed that the bilingual group distinguished the English from French videos whereas the monolinguals did not. This suggests that monolinguals and bilinguals could use different processing mechanisms to decode visual speech. These results concerned language discrimination in a story. Do bilinguals process visual information for phonemes that do not exist in their phonological inventory as monolinguals do? The present study examined whether linguistic experience during early childhood affects the visual processes involved in non-native phoneme identification in audiovisual speech.

Children who grow up in a bilingual environment (or are exposed to a foreign language very early in life) seem to be particularly sensitive to native and non-native sounds. Byers-Heinlein et al. (2010) presented data indicating that newborns who were prenatally exposed to one language (i.e., English monolingual mother or Tagalog monolingual mother) preferred their mothers' language. If bilingual mothers spoke both languages during pregnancy, the newborns had no preference for either language. Furthermore, if the bilingual mothers spoke English and Chinese during pregnancy, the neonates had no preference for a language spoken during the pregnancy (English) or a new one (Tagalog). This suggests that “bilingual” neonates could process speech sounds differently (Burns et al., 2003; Kuhl et al., 2003) and lead to differences in the neural structure in the auditory cortex as adults (Ressel et al., 2012).

In sum, visual information on the face movements of the speaker seems to be extremely useful to decode speech. Monolinguals and bilinguals seem to process visual language differently, at least during the first year of life. Are these differences still present during adulthood? Do they use the same processing mechanisms to decode visual speech for phoneme identification? Do monolinguals and bilinguals use visual information to overcome phonological deafness? We conducted an experiment with monolingual and bilingual participants to answer these questions. The participants had to discriminate a Bengali plosive dental-retroflex contrast (/t/-/ʈ/) that does not exist in any of the participants' languages. The dental /t/ phoneme exists in all the participants' phonological inventories whereas the retroflex counterpart does not exist in any of them. The retroflex consonant we used is a coronal consonant where the tongue has a curled shape and is articulated between the alveolar ridge and the hard palate. The retroflex feature is articulated further back of the vocal tract than the dental. Moreover, during the articulation of the dental the tongue is apparent after the burst release. This means that the dental-retroflex contrast is both auditorily and visually salient. The recordings were presented in an audio-only condition to examine whether the two groups differed in their abilities to discriminate between native and non-native phonemes. We also presented the Bengali recordings with their corresponding videos in an audiovisual presentation to investigate whether the visual information on the speaker's face movements contributed to overcome the difficulties in phoneme identification for the non-native contrast. Since monolinguals and bilinguals process visual speech differently during early childhood, it is likely that their abilities for visual speech processing is also different as adults.

## Methods

### Participants

Information on the participants' linguistic experience was collected with an adapted version of the “Language Experience and Proficiency Questionnaire” (Marian et al., 2007). There were 47 bilinguals. Although we did not directly study the issue of early vs. late bilingualism, we selected the bilingual participants on the basis of an early exposure to two languages and a high proficiency with them. There were 24 Catalan-Spanish bilinguals (4 men and 20 women; mean age = 20 years). They have all been exposed to both languages very early in childhood. Mean age of acquisition of Spanish and Catalan was 11 and 14 months, respectively. Ten learnt Spanish and Catalan at home. Seven have always been exposed to Spanish but lived in a Catalan environment and seven have been exposed to Catalan from birth but learnt Spanish in nursery school. They were students at the University Pompeu Fabra (Barcelona, Spain). There were 23 bilinguals of different languages (8 men and 15 women; mean age = 19.6 years). These bilinguals spoke French and another language from birth: English (4 participants), German (4 participants), Italian (4 participants), Spanish (3 participants), Malgash (2 participants), Portuguese (2 participants), Arab (2 participants), Polish (2 participants). They have all been exposed to both languages from birth. They spoke French because they lived in Grenoble from birth and the other language was their parents' native mother tongue. They were balanced bilinguals with equivalent proficiency in both languages (Marian et al., 2007). They were students at the University of Grenoble or at the Cité Scolaire Internationale which is an international school in Grenoble where only bilinguals can attend. The monolingual group consisted of 47 French native speakers (8 men and 39 women; mean age = 22 years). They all learnt English as a second language in middle and high school but their proficiency was very poor. They had no experience in a foreign country of more than 1 month. They were students at the University of Grenoble and received course credit for participation.

### Material

**Figure 1:**
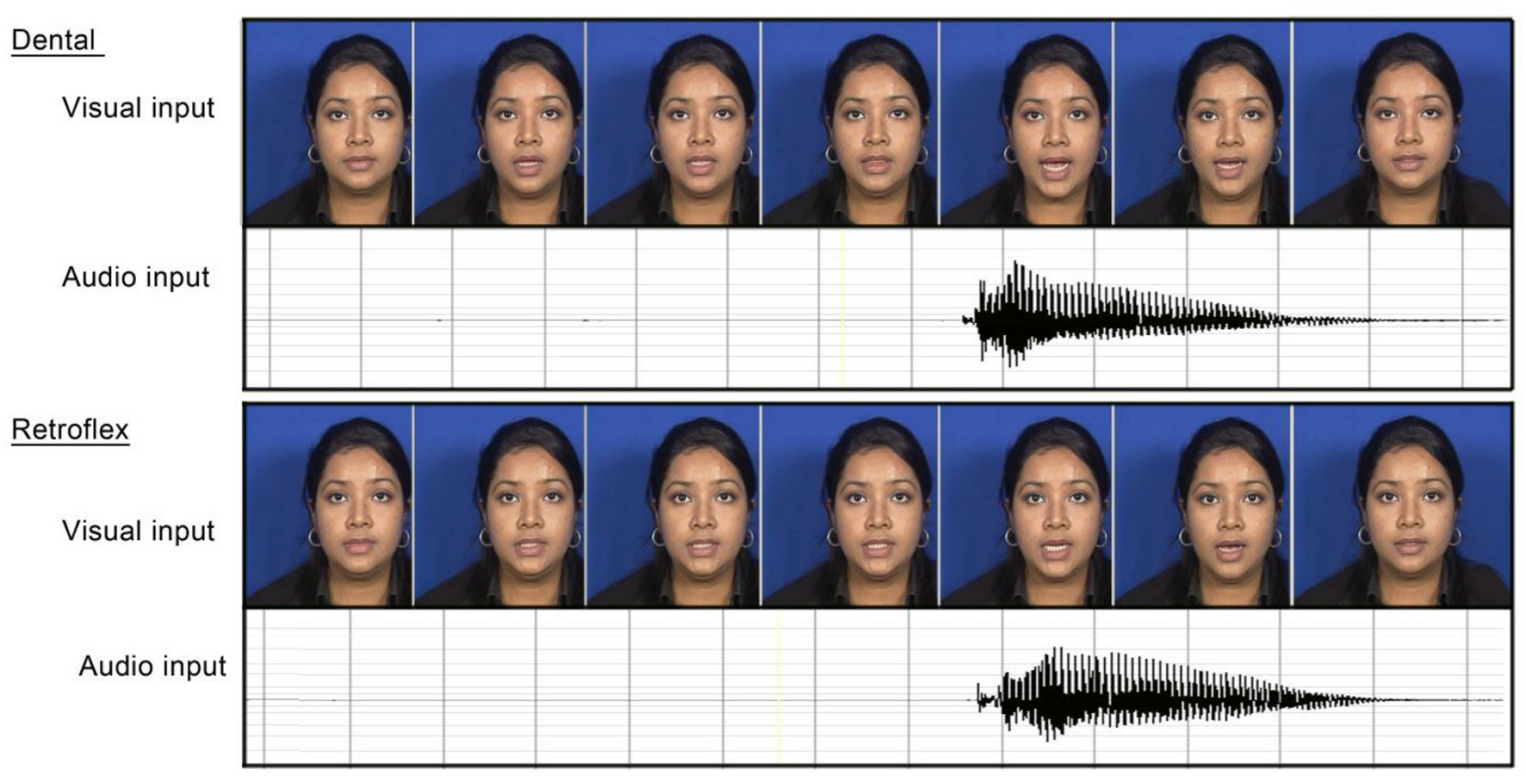
**Description of the audio and visual characteristics of the native (/ta/) and non-native (/ʈa/) stimuli**.

We recorded 20 tokens of two Bengali syllables that differed in the plosive dental/retroflex phonological contrast (/ta/ and /ʈa/). This contrast does not exist in any of the languages spoken by the participants. The plosive dental phoneme /ta/ exists in all of the participants' languages, whereas the retroflex plosive /ʈa/ does not. The stimuli were recorded in a sound proof room by a native female Bengali speaker from Bangladesh. We presented the full face of the speaker with a blue background (see Figure 1). The recordings were done with a tri-CCD SONY DXC-990P camera and an AKG C1000S microphone. They were converted into AVI video files (PAL format, 25 img/s) and were segmented manually with the DpsRealitysoftware. Each /ta/ and /ʈa/ sequence began and ended with the speaker with the mouth closed. We selected 11 tokens of each sequence out of the 20 recordings. Figure 1 describes the audio and visual characteristics of the stimuli for a dental and a retroflex token.

### Procedure

The experiment was conducted with an ABX paradigm. It was programmed with Eprime® software2.0 (Psychology Software Tools, Inc.). Ten tokens of /ta/ and 10 of /ʈa/ were used as X stimuli and one token of each syllable as references for A and B, for a total of 20 trails. We presented the A token, then the B token and finally the X stimulus. The participants were instructed to press one key of the keyboard if the X stimulus presented the same syllable as the A token or another key if the X stimulus presented the same syllable as the B token. The correct responses were equally distributed between both hands. The participants were instructed to respond as quickly and as accurate as possible. The X stimuli were presented randomly within a block. The identity (i.e., dental or retroflex) of the A and B tokens were fixed all along the experiment and controlled between participants. In the audio-only condition (A hereafter) we presented the audio track of the stimuli and the computer screen displayed an image with a still face of the speaker. In the audiovisual condition (AV hereafter) we presented the same audio track but the screen displayed the video with the moving face of the speaker. The experiment consisted of two blocks that were counterbalanced between participants; one for each presentation modality. The participant either heard (A-only) or saw and heard (AV condition) the Bengali sequences, or vice-versa.

The task was displayed by a Monitor LCD Dell (17 inches). The video stimuli were presented at 25 frames/s with a resolution of 720 × 576 pixels. The auditory component of the stimuli was provided at a 44100 Hz sampling rate by two SONY SRS-88 speakers located on both sides of the screen. In both conditions, the participants were instructed to respond on the basis of what they *perceived*, without referring to the auditory or visual modalities. We recorded correct responses (Accuracy) and the reaction time of the correct responses (RT). The participants were tested individually in a quiet room. They sat 40 cm away from the screen and the sound level was set to a comfortable level. Before the task, we made sure the participants understood the task by a short training session in A-only and AV presentation of Vietnamese consonant-vowel syllables. The experiment lasted approximately 30 min (questionnaires, instructions, and experimentation).

## Results

The results were analyzed using linear mixed effects models (Bates, 2005; Baayen et al., 2008), which simultaneously take participant and item variability into account. These analyses were performed using the software R (R Development Core Team, Bates and Maechler, 2009) with the package lme4 (Bates and Maechler, 2009). The statistical analyses were performed on Accuracy and Reaction time with Group (Monolingual, Bilingual), Modality (Audio-only, Audiovisual), and Phoneme (Native, Non-Native) as factors.

### Accuracy

The results for Accuracy are presented in Table 1.

**Table 1:** **Accuracy values (%) and standard errors (in brackets) for bilinguals and monolinguals in the A-only and AV presentation modalities for native and non-native phonemes**.

	**A-only**		**AV**	
	**Native**	**Non-native**	**Native**	**Non-native**
Monolingual	62 (7)	55 (7)	88 (5)	75 (6)
Bilingual	56 (7)	59 (7)	76 (6)	66 (7)

**Figure 2:**
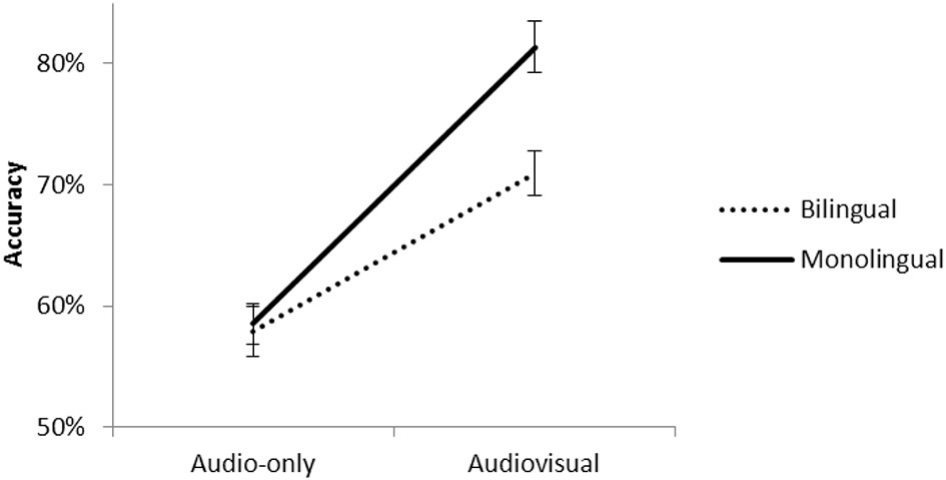
**Accuracy for monolinguals and bilinguals in the audio-only and audiovisual presentations of the Bengali phonemes**.

The analysis revealed no significant main effects (Table A1). In contrast, the interactions between the factors were significant. The three way interaction was not significant, *t*_(3757)_ = −1.14, *p* = 0.25. The interaction between Modality and Group reached significance, *t*_(3757)_ = 3.13, *p* < 0.001. Figure 2 presents the percentage of correct responses for monolinguals and bilinguals for the audio-only and audiovisual presentations.

Pairwise comparisons revealed that both groups had better scores in the AV than A presentations [monolinguals, *t*_(1879)_ = 11.34, *p* < 0.001; bilinguals, *t*_(1879)_ = 6.07, *p* < 0.001]. However, the “Audiovisual benefit” (AV score—A score) was higher for monolinguals (22%) than bilinguals (13%), *t*_(1878)_ = 2.71, *p* < 0.01. In the AV condition, monolinguals had higher scores than bilinguals, *t*_(1879)_ = 3.50, *p* < 0.001. In contrast, there were no group differences in the A condition, *t*_(1879)_ = 0.22, *p* = 0.82. To test for phonological deafness, we compared the accuracy scores for the non-native retroflex phoneme in the audio-only condition with chance level (50%) for each group. The scores for monolinguals (54.8%) did not reach significance [*T*_(1, 46)_ = 1.85, *p* = 0.06] and was slightly above chance (59.3%) for bilinguals, *T*_(1, 46)_ = 3.04, *p* < 0.001.

**Figure 3:**
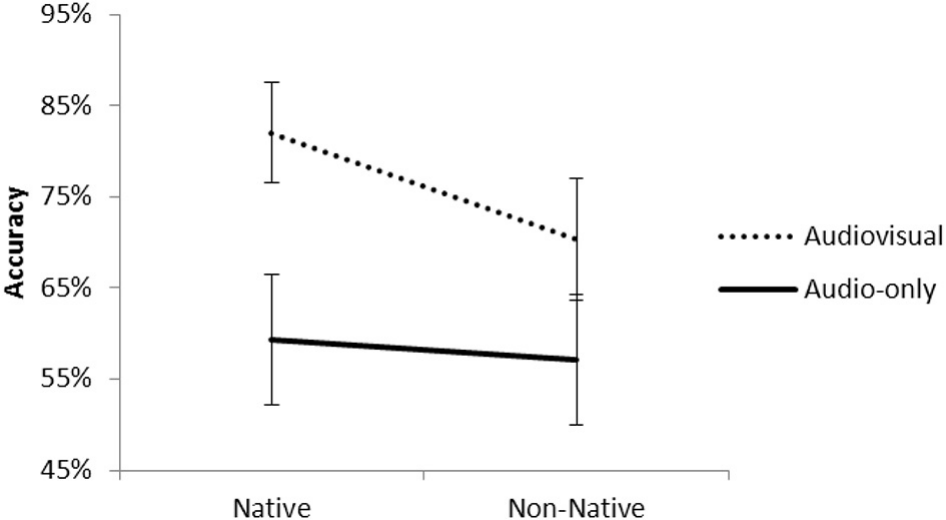
**Accuracy for native and non-native phonemes in the audio-only and audiovisual presentations**.

The interaction between Modality and Phoneme was significant, *t*_(3757)_ = 3.08, *p* < 0.01. Figure 3 presents the percentage of correct responses for native and non-native phonemes for the audio-only and audiovisual presentations.

Pairwise comparisons revealed that for both phonemes the scores improved in the AV presentation with respect to the A presentation [native, *t*_(1879)_ = 11.83, *p* < 0.001; non-native, *t*_(1879)_ = 6.20, *p* < 0.001]. However, the “Audiovisual benefit” (AV score—A score) was greater for the native phonemes (22%) than non-native phonemes (13%), *t*_(1878)_ = 3.23, *p* < 0.001. In the audiovisual presentation, the scores for the native phonemes were higher than non-native phonemes, *t*_(1879)_ = 4.90, *p* < 0.001. In contrast, there were no differences between the phonemes in the audio-only condition, *t*_(1879)_ = 0.84, *p* = 0.40.

**Figure 4:**
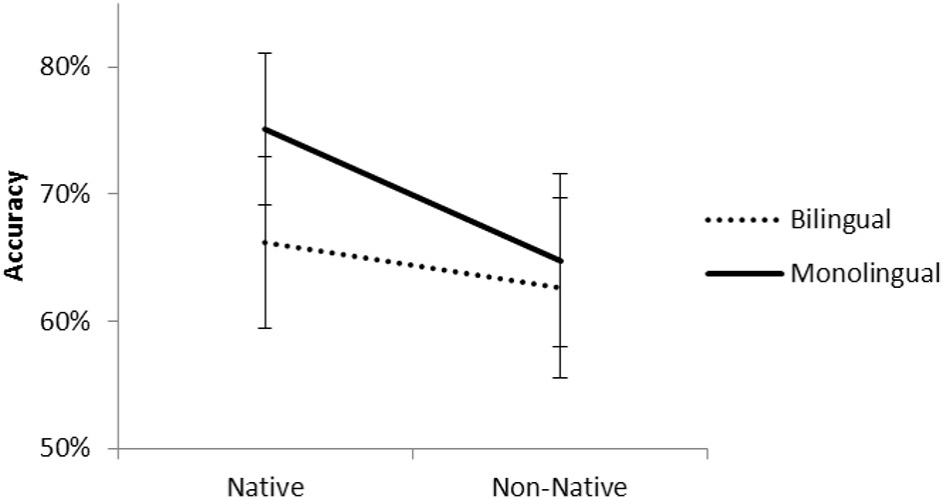
**Accuracy for native and non-native phonemes for monolinguals and bilinguals**.

The interaction between Group and Phoneme was also significant, *t*_(3757)_ = 2.42, *p* < 0.01. Figure 4 presents the percentage of correct responses for native and non-native phonemes for monolinguals and bilinguals.

Pairwise comparisons revealed that the scores for the two groups were equivalent for the non-native phonemes, *t*_(1879)_ = 0.64, *p* = 0.52. In contrast, for the native phonemes the scores for monolinguals were higher than bilinguals, *t*_(1879)_ = 2.40, *p* < 0.01. For monolinguals, the scores for native phonemes were higher than the non-native ones, *t*_(1879)_ = 4.34, *p* < 0.001. For bilinguals, the scores were equivalent for the two kinds of phonemes, *t*_(1879)_ = 1.61, *p* = 0.10^1^.

### Reaction time

RTs faster than 300 ms and slower than 3000 ms were excluded from the analysis (4.41% of the data). The results for Reaction Time of correct responses are presented in Table 2.

**Table 2:** **Reaction time values (ms) and standard errors (in brackets) for bilinguals and monolinguals in the A-only and AV presentation modalities for native and non-native phonemes**.

	**A-only**		**AV**	
	**Native**	**Non-native**	**Native**	**Non-native**
Monolingual	1428 (70)	1498 (73)	1251 (62)	1303 (63)
Bilingual	1495 (70)	1573 (72)	1385 (73)	1501 (72)

**Figure 5:**
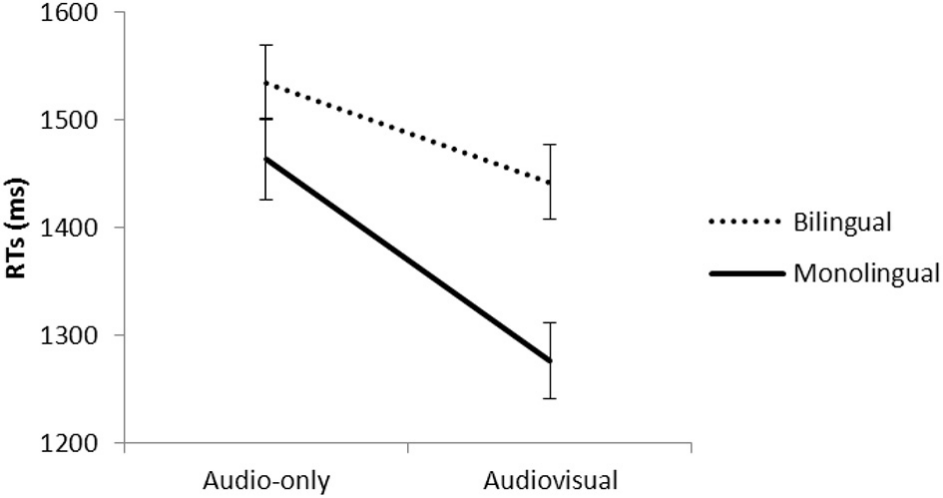
**Mean reaction times (ms) on correct responses for monolinguals and bilinguals in the audio-only and audiovisual presentations of the Bengali phonemes**.

The analysis (Table A2) revealed that monolinguals were globally faster than bilinguals, *t*_(3591)_ = −2.45, *p* < 0.01. The Modality effect almost reach significance, *t*_(3591)_ = −1.82, *p* = 0.06, indicating that correct responses were faster in the AV than A presentations. Responses for native phonemes were faster than non-native phonemes, *t*_(3591)_ = −2.31, *p* < 0.05. The three way interaction was not significant, *t*_(3757)_ = 0.87, *p* = 0.38. The interaction between Modality and Group was significant, *t*_(3591)_ = −2.75, *p* < 0.001. Figure 5 presents the reaction time for monolinguals and bilinguals for the audio-only and audiovisual presentations.

Pairwise comparisons revealed that both groups respond faster in the audiovisual presentation with respect to the audio-only presentation [monolinguals, *t*_(1822)_ = −10.22, *p* < 0.001; bilinguals, *t*_(1822)_ = −4.44, *p* < 0.001]. The “Audiovisual benefit” (A RT—AV RT) was numerically greater for monolinguals (150 ms) than bilinguals (104 ms), but the difference did not reach significance, *t*_(1878)_ = −1.00, *p* = 0.31. In the audiovisual presentation, monolinguals were faster to respond than bilinguals, *t*_(1791)_ = −3.20, *p* < 0.001. In contrast, there were no group differences in the audio-only condition, *t*_(1791)_ = −1.39, *p* = 0.16.

## Discussion

The aim of this study was to examine whether monolinguals and bilinguals—who had a different linguistic experience during early childhood and after—take advantage of the visual information on the speaker's face movements when they have to identify phonemes that do not exist in their native language/s. Monolingual and bilingual participants had to identify Bengali phonemes that differ on a dental/retroflex consonant contrast that does not exist in any of the languages they speak. The phonemes were presented in an audiovisual condition where a video presented the visual information of the speaker producing the phonemes and their corresponding sound. In the audio-only condition the participants were presented the same stimuli but without the visual information. The results indicated that in the audio-only presentation monolinguals and bilinguals had similar difficulties in discriminating the Bengali retroflex phoneme. In the audiovisual condition instead, both groups took advantage of the visual information on the speaker's face movements to identify the non-native retroflex phoneme. They could overcome—at least partially- the difficulties experienced in the audio-only condition. The visual information not only enhanced identification but also accelerated phoneme processing. The results also point to visual processing differences between the two groups, since in the AV condition monolinguals were more accurate and faster than bilinguals. Furthermore, the “audiovisual benefit” was greater for monolinguals than bilinguals indicating that linguistic exposure to more than one language may affect the visual processing of non-native phonemes.

Monolinguals and bilinguals had similar accuracy scores and reaction times in the audio-only condition. This suggests that when the participants heard the Bengali /ʈ/ retroflex phoneme—that does not exist in their phonological repertoire—they assimilated it to /t/ that exists in the languages they speak. This assimilation phenomenon leads to serious difficulties in phoneme identification and occurs because they cannot process the auditory relevant cue that distinguishes the two phonemes (Best et al., 2001). This is in agreement with previous research showing that early bilinguals can have monolingual-like performance during unfamiliar phoneme perception (Pallier et al., 1997; Sebastián-Gallés and Soto-Faraco, 1999). Also, Von Holzen and Mani (2012) showed that French-German preschooler bilinguals failed at discriminating Salish consonants. Moreover, Navarra and Soto-Faraco (2007)'s study indicated that in an audio-only condition Spanish-Catalan bilinguals who were Spanish dominant could not discriminate the Catalan phonemes /e/ and /ε / (only /e/ exists in Spanish). This suggests that the particular sensitivity bilinguals have during the first year of life does not necessarily extend to non-native phonemes later in life. Even if several studies on infant perception showed more phoneme sensitivity in bilinguals, perceptual development keeps changing after the first year of life (Sundara et al., 2006). Our findings are in line with this idea, since in the audio-only condition the bilinguals did not exhibit a particular phoneme identification advantage with respect to monolinguals.

The main contribution of the present study concerns the visual component of non-native phoneme identification processes. All the participants could exploit the visual cues that distinguish retroflex /ʈ/ from dental /t/ in the audiovisual condition. Even if the retroflex feature does not exist in the participants' phonological inventory, the visual differences between the two consonants are salient enough to identify them. So the visual information on the speakers' facial movements played an important role in overcoming, at least partially, the phoneme identification difficulties the two groups experienced in the audio-only condition. The audio-visual benefit was higher for the native than the non-native phoneme. To our knowledge, the only study on audiovisual phoneme perception conducted with bilinguals is the one carried out by Navarra and Soto-Faraco (2007). As mentioned above, Spanish-Catalan bilinguals who were Spanish dominant could not discriminate the Catalan phonemes /e/ and /ε / in an audio-only condition. In the AV condition, they were able to overcome the difficulties in phoneme identification, as in our experiment. What we do not know from Navarra and Soto-Faraco (2007) is whether these bilingual participants performed differently than monolinguals because the authors did not include a monolingual group in their study. Our research provides an answer to this question.

The results of the present study revealed that monolinguals and bilinguals do not take the same advantage of visual information on phoneme identity. In the AV condition, monolinguals were more accurate than bilinguals. In addition, the “Audiovisual benefit”—i.e., the accuracy score increase from A to AV—was 9% higher for monolinguals than bilinguals. We also observed that perceiving the speaker's speech gestures accelerated phoneme processing for both groups but again, the bilingual group seemed less sensitive to visual information. The acceleration was more pronounced in monolinguals than bilinguals. The difference in “Audiovisual benefit”—i.e., the decrease in reaction time—between the groups was of 46 ms but the difference failed to reach statistical significance. This is consistent with Sebastian-Gallés et al. (2012)'s study suggesting that monolinguals and bilinguals use different processing mechanisms to decode visual speech. However, the latter was conducted with 8 month-old infants and concerned language discrimination tasks and not phoneme identification, so we do not know which component of visual speech processing is responsible for these differences.

Studies on non-native phoneme identification/discrimination showed that early exposure to several languages delays perceptual narrowing and could lead to a better performance for non-native phonemes (Burns et al., 2003; Kuhl et al., 2003; Byers-Heinlein et al., 2010). Our findings do not confirm this “Bilingual advantage.” Being exposed to several languages may delay perceptual narrowing during infancy, but it does not necessarily lead to a benefit for identifying non-native phonemes in adulthood. In fact, the facilitation bilinguals benefit from during childhood may result in a processing “cost” during adulthood. For example, Costa et al. (2000) provided reaction time data indicating that bilinguals were slower than monolinguals in picture naming tasks involving lexical access.

Another possibility is that bilinguals take less advantage of visual speech than monolinguals because they are neurally better “equipped” to process auditory information. Golestani et al. (2007) measured the volume of Heschl's gyrus in French participants who learnt a Hindi dental/retroflex consonant contrast “fast” and “slow.” Heschl's gyrus is located in the auditory cortex and is the first cortical area that receives auditory information coming from the peripheral auditory system. They observed that the fast participants had bigger Heschl's gyrus volumes than the slower ones. According to the authors the bigger Heschl's gyrus volumes in fast participants could make them have a better temporal representation of sounds. This would be extremely useful to discriminate the rapid acoustic transitions that we observe in many consonants and thus enhance discrimination abilities for the dental/retroflex Bengali consonant contrast. Furthermore, Ressel et al. (2012) measured monolinguals' (Spanish) and bilinguals' (Spanish-Catalan) volume of Heschl's gyrus. They provided evidence indicating that bilinguals have larger Heschl's gyri than monolinguals. The voxel-based morphometry data for the left Heschl's gyrus indicated that the gray matter volumes were more important in bilinguals than monolinguals. The positive correlation between larger Heschl's gyri and the ability to perceive non-native phonemes suggests that bilinguals would have better auditory capacities to discriminate the Bengali phonemes and would rely less on visual speech. Although this hypothesis is very appealing, it is not supported by our results, since monolinguals and bilinguals had equivalent scores in the audio-only condition for the discrimination of the Bengali dental/retroflex consonant contrast.

The fact that monolinguals were more efficient than bilinguals in the audiovisual condition could also reveal another “bilingual cost” that may have nothing to do with phoneme identification *per se* but with the visual processing of the speaker's face. To decode visual speech in face-to-face communication we have to process the speaker's face. We have to do a configural analysis to locate the mouth with respect to the eyes, nose, etc. We will then be able to process the movements that transmit the relevant information on phoneme identity. This means that there could be a link between face processing and speech perception. If so, would the bilingual perceptual narrowing pattern observed for visual speech (Sebastian-Gallés et al., 2012) result, or be related to, perceptual narrowing in face processing? From a developmental perspective, face processing and phoneme identification are both important for early communication. Faces can be seen as providing an early channel of communication prior to the onset of gestural or oral language between infant and caretaker (Pascalis et al., 2014). The idea of a link between face processing and lip-reading is not new. In 1986, Bruce and Young's face recognition model already included an optional *facial speech analysis* module that categorized oro-facial movements (Bruce and Young, 1986).

Moreover, some studies suggested that bilingual exposure could lead to changes in brain organization and affect face perception and spatial localization tasks that are linked to hemispheric asymmetry (Sewell and Panou, 1983). More recently, Haussman et al. (2004) investigated hemispheric specialization differences between German monolinguals and German-Turkish bilinguals during linguistic and face-discrimination tasks. The results indicated that bilinguals do not have the same left visual field advantage than monolinguals during face discrimination. Bilinguals' reaction times were longer than monolinguals' when the faces were presented in the left visual field, indicating a difference in cortical organization for face processing between the two populations. These temporal differences are consistent with our study. We also observed that bilinguals' reaction times were slower than monolinguals' in the audiovisual condition. On this basis, and if bilinguals and monolinguals process faces differently, it can have an impact on their abilities to process visual speech. Further research has to be done, of course, to investigate whether the bilinguals' lower phoneme identification scores and higher RTs with respect to monolinguals in the audiovisual presentation could be due to differences in face processing.

To conclude, linguistic experience has an impact on the way we process visual speech. Monolinguals are more accurate and faster than bilinguals to process the speaker's articulatory gestures. This gives them an advantage when having to identify phonemes. Indeed, the former benefit more from visual information than the latter with respect to audio-only communication.

### Conflict of interest statement

The authors declare that the research was conducted in the absence of any commercial or financial relationships that could be construed as a potential conflict of interest.

## Footnotes

^1^We conducted an additional analysis to see whether the participants' performance increased in the second block: A then AV and AV then A. We did a LMM analysis with group (monolinguals, bilinguals) and presentation order (A then AV; AV then A) as factors. The results did not yield a significant block effect, *t*_(3758)_ = 1.44, *p* = 0.14.

## Appendices

**Table A1:** **Statistical analyses on accuracy values for group (bilinguals, monolinguals), modality (A-only, AV), and phoneme type (native, non-native)**.

**Factors**	***t*-value**	**Pr(>|*t*|)**
Group	−1.073	0.2835
Modality	2.343	0.0192
Phoneme	−0.603	0.5465
Group^*^Modality	3.287	0.001
Group^*^Phoneme	2.286	0.0223
Modality^*^Phoneme	3.215	0.0013
Group^*^Modality^*^Phoneme	−1.143	0.253

* *Indicates interaction*.

**Table A2:** **Statistical analyses on Reaction time values for group (bilinguals, monolinguals), modality (A-only, AV), and phoneme type (native, non-native)**.

	***t*-value**	**Pr(>|*t*|)**
Group	−3.33	0.0009
Modality	−2.34	0.0191
Phoneme	−3.03	0.0024
Group^*^Modality	−3.02	0.0025
Group^*^Phoneme	1.15	0.2489
Modality^*^Phoneme	−0.36	0.716
Group^*^Modality^*^Phoneme	0.87	0.386

* *Indicates interaction*.
